# Proteases from the Regenerating Gut of the Holothurian *Eupentacta fraudatrix*

**DOI:** 10.1371/journal.pone.0058433

**Published:** 2013-03-07

**Authors:** Nina E. Lamash, Igor Yu Dolmatov

**Affiliations:** 1 A.V. Zhirmunsky Institute of Marine Biology of the Far Eastern Branch of the Russian Academy of Sciences, Vladivostok, Russia; 2 School of Natural Sciences, Far Eastern Federal University, Vladivostok, Russia; University of Oxford, United Kingdom

## Abstract

Four proteases with molecular masses of 132, 58, 53, and 47 kDa were detected in the digestive system of the holothurian *Eupentacta fraudatrix*. These proteases displayed the gelatinase activity and characteristics of zinc metalloproteinases. The 58 kDa protease had similar protease inhibitor sensitivity to that of mammalian matrix metalloproteinases. Zymographic assay revealed different lytic activities of all four proteases during intestine regeneration in the holothurian. The 132 kDa protease showed the highest activity at the first stage. During morphogenesis (stages 2–4 of regeneration), the highest activity was measured for the 53 and 58 kDa proteases. Inhibition of protease activity exerts a marked effect on regeneration, which was dependent on the time when 1,10-phenanthroline injections commenced. When metalloproteinases were inhibited at the second stage of regeneration, the restoration rates were decreased. However, such an effect proved to be reversible, and when inhibition ceased, the previous rate of regeneration was recovered. When protease activity is inhibited at the first stage, regeneration is completely abolished, and the animals die, suggesting that early activation of the proteases is crucial for triggering the regenerative process in holothurians. The role of the detected proteases in the regeneration processes of holothurians is discussed.

## Introduction

The extracellular matrix (ECM) plays an important role in the vitality of multicellular organisms [1]. Its formation and ability to function depends on a large number of proteins (collagens, integrins, laminins, etc.), cellular surface receptors, and proteases. The ECM in each tissue or organ contains a unique set of factors that influence cell-cell interactions, cell differentiation and migration, thus making it very important in regulating embryonic development, growth, regeneration, and tumorigenesis [2], [3], [4]. The basis for ECM remodeling is provided by specific proteases, such as matrix metalloproteinases (MMPs) [5] and aminopeptidases [6]. These enzymes are able to degrade all known types of ECM proteins and, hence, play an important role in embryonic development and regeneration; in the resorption and remodeling of tissue; in the migration, differentiation and proliferation of cells in both vertebrate and invertebrate animals; and in invasion and tumor metastasis [7], [8].

The MMPs were first found in amphibians [9]. The tails of tadpoles, which are destroyed during metamorphosis, were shown to contain an enzyme capable to degrade the collagen I. Later on, the MMP were found to play a significant role in the regulation of development and metamorphosis in different animals [3], [10], [11], [12], [13], [14]. Diverse aspects of ECM remodeling and MMP activities during physiological processes (development and regeneration) and diseases in mammals have being investigated intensively [11], [15], [16], [17], [18]. The role of ECM remodeling in invertebrate regeneration has been studied to a lesser degree. Page-McCaw [12] cited only five species in which the connective tissue and an MMP were studied: the fruit fly *Drosophila melanogaster*, nematode worm *Caenorhabditis elegans*, *Hydra vulgaris* and *H. magnipapillata*, and sea urchin *Strongylocentrotus purpuratus*.

Among echinoderms the most well-studied model objects are different species of sea urchins. In these animals the MMP were first found in developing embryos [19], [20], [21], [22]. They are supposed to regulate the processes of gastrulation and hyaline layer development, as well as the growth of spicules in sea urchins. An analysis of genome in the sea urchin *S. purpuratus* revealed numerous genes of metalloproteinases [23].

Echinoderms are convenient model organisms for the study of various aspects of regeneration. These animals exhibit high restoration capability and are able to regenerate both external appendages and internal organs [24], [25]. Among echinoderms, the regeneration processes have been best studied in representatives of the class Holothuroidea, the sea cucumbers. Regeneration in these animals is often accompanied by connective tissue remodeling. The regeneration of muscles, in particular, involves the formation of a connective-tissue cord at the place of injury into which myogenic cells migrate [25], [26], [27], [28]. The reorganization of the ECM during gut regeneration in holothurians has also been reported [29], [30], [31].

Some holothurian species are capable of evisceration, which is a unique kind of autotomy. In response to various irritants, holothurians eject the gut and then regenerate it within a fairly short period of time. The new intestine is formed along the margin of the intestinal mesentery to which the gut was attached to the holothurian body wall. Early during the regeneration process, the torn edge of the mesentery is transformed into a connective-tissue cord to which cells migrate, subsequently forming the intestinal lining [24], [32], [33]. Two types of evisceration are known in holothurians [27], [32], [33]. The holothurians of the order Aspidochirotida eject the intestine through anal opening. Only the middle part of digestive tube is removed in this case; both the anterior (pharynx and esophagus) and posterior (cloaca) regions are retained. Regeneration after the evisceration includes only the transformation of intestinal mesentery and the retained broken ends of esophagus and cloaca. In the members of the order Dendrochirotida evisceration is performed through the anterior end of the body. During the evisceration the entire digestive system (excluding cloaca) and the oral complex of organs (the aquapharyngeal complex, AC) are rejected. The AC plays an important role in vital activities of holothurians, as, besides the proximal parts of intestinal tube (pharynx and esophagus), it comprises nerve ring, water-vascular ring canal and hemal ring. These are important integrating structures uniting radially located parts of corresponding systems of organs. Regeneration of Dendrochirotida is interesting, first of all, because during the evisceration all tissues of entodermal origin are removed and regeneration of gut lining takes part at the expense of mesothelium [34]. Moreover, the mechanisms of regeneration are more diverse, as the latter comprises development of not only digestive system, but also musclular, water-vascular, hemal and nerve systems. The primordium of AC is mostly built of connective tissue, which demonstrates the great role of ECM remodeling not only in regeneration of the gut, but rather the entire anterior end of the holothurian.

Despite the important role of ECM remodeling in regeneration, the mechanisms involved in this process in echinoderms have virtually not been studied. The available 3 papers deal only with holothurians of the order Aspidochirotida [29], [35], [36]. It was shown that during regeneration after evisceration in *Holothuria glaberrima*, the collagen content in the gut primordium is decreased, whereas the activity of MMPs is increased [29]. Four proteins with gelatinase activity that varied during regeneration were found in this species. The use of MMP inhibitors retarded gut regeneration. However, the paper of Quiñones et al. [29] did not describe the biochemical properties of proteases, thus their belonging to the family of matrix metalloproteinases was not corroborated adequately enough. Moreover, the activity of proteases was examined in respect to only two substrates, namely gelatin and casein. Recent molecular genetic studies on aspidochirotids *H. glaberrima* and *Apostichopus japonicus* revealed the activity of four genes (*MMP-11, MMP-14, MMP-15,* and *MMP-17*) coding for the corresponding metalloproteinases of the ECM; however, the proteins themselves were not identified [35], [36]. Phylogenetic analyses showed that different ancestral genes were differently amplified in echinoderms and vertebrates, thus the MMP families in these two groups of animals cannot be entirely homologous to each other [23]. Thus, additional studies of biochemical properties and substrate specificity of corresponding proteins are required to corroborate that the genes revealed in holothurians are really MMP-coding.

**Figure 1:**
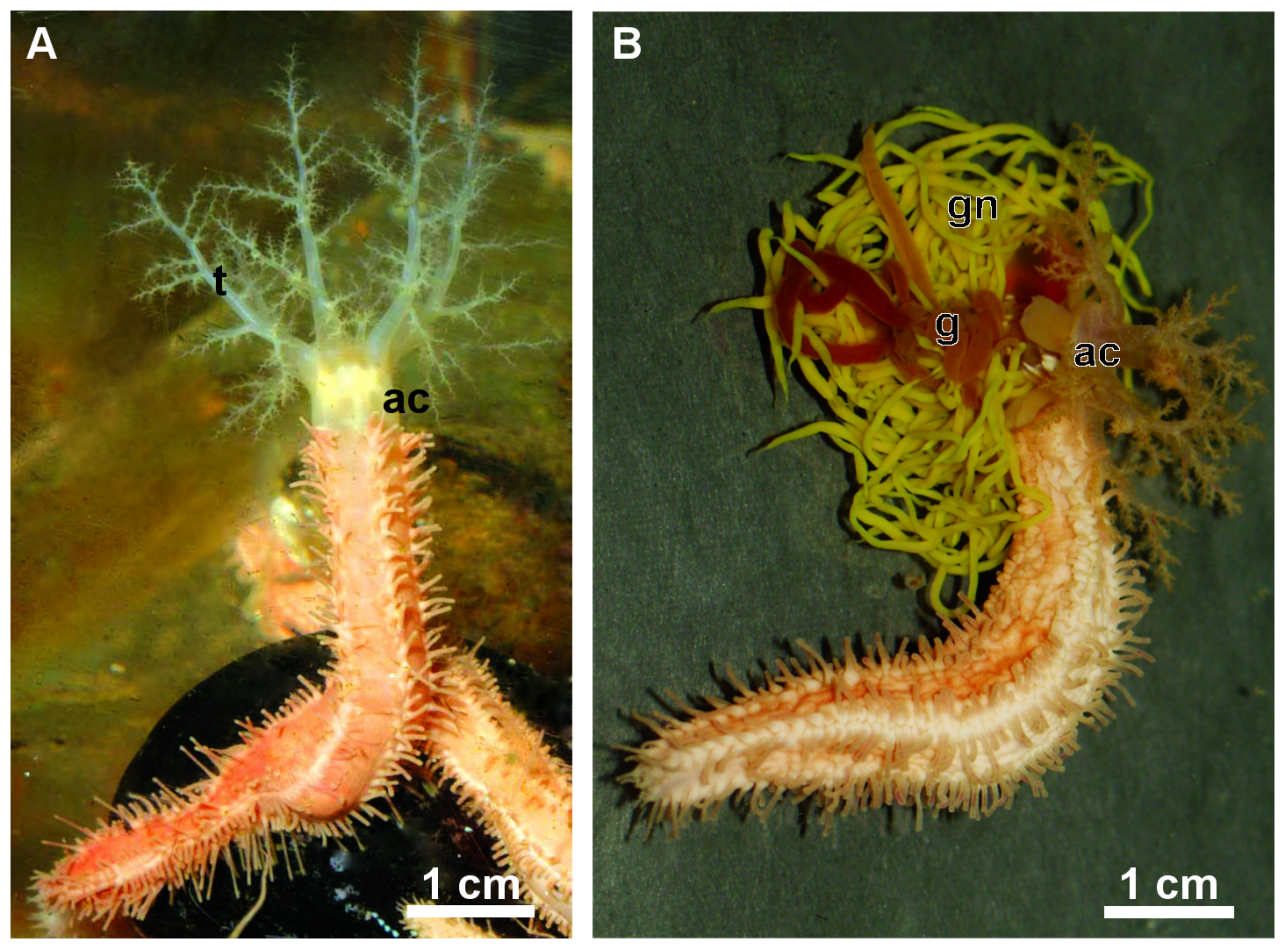
The holothurian *Eupentacta fraudatrix.* (A) General view of a holothurian with exposed aquapharyngeal complex and tentacles. (B) An animal with eviscerated aquapharyngeal complex, gut and gonad tubules. Abbreviations: ac, aquapharyngeal complex; g, gut; gn, gonad tubules; t, tentacles.

**Figure 2:**
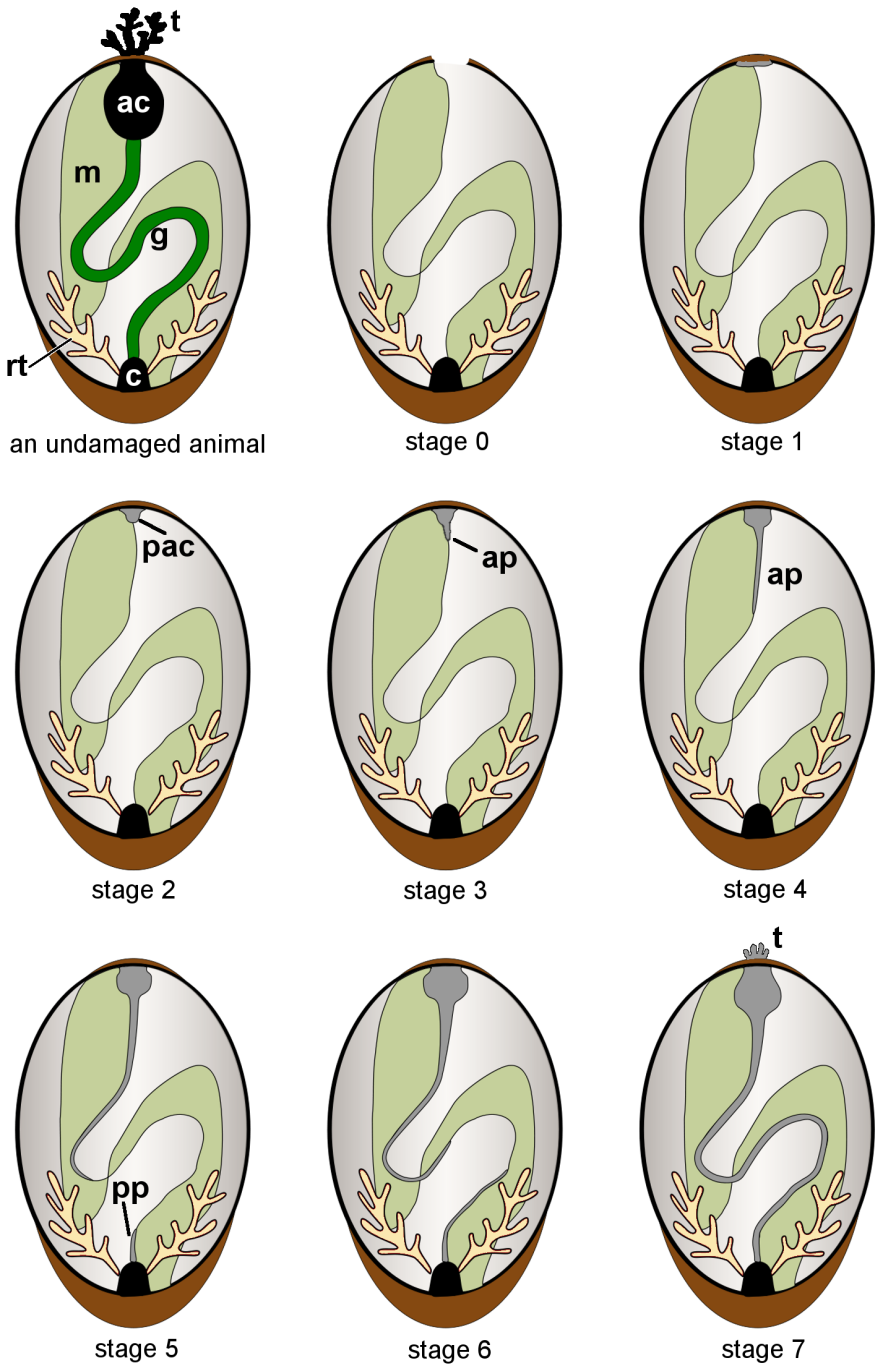
Scheme of the consecutive stages of internal organ regeneration in the holothurian *Eupentacta fraudatrix.* An undamaged animal. It contains aquapharyngeal complex with tentacles, gut with mesentery, gonads (not shown) and organs of respiration (respiratory trees). Stage 0 (just after evisceration). The animal after evisceration contains only cloaca, respiratory trees and intestinal mesentery. On the anterior end of the animal the body wall is ruptured. Stage 1 The rupture on the anterior end is closed by thrombus and connective tissue. Stage 2 A connective tissue is thickening; the rudiment of AC is formed at the anterior end of the holothurian. Stage 3 A rod-like thickening, the anterior gut rudiment, begins growing backward from AC rudiment, along the torn edge of mesentery. Stage 4 The anterior gut rudiment elongates. Development of AC is continued. On this stage nerve ring and water-vascular ring canal are formed. Stage 5 The posterior rudiment becomes noticeable. Growth of AC and the anterior gut rudiment is continued. Stage 6 The main structures of AC develop, and the AC increases in size. The anterior gut rudiment continues growing backward, along the mesenteric edge, and reaches the middle of the body. Stage 7 The anterior and posterior rudiments fuse to each other to restore the entire gut. Abbreviations: ac, aquapharyngeal complex; ap, anterior primordium; c, cloaca; g, gut; m, mesentery; pac, primordium of the aquapharyngeal complex; pp, posterior primordium; rt, respiratory trees; t, tentacles.

The sea cucumber *Eupentacta fraudatrix* is belongs to the order Dendrochirotida. In our opinion, this species is an interesting model object to study different aspects of regeneration. This species ejects viscera through the anterior end of the body (Fig. 1). As a result, most of the internal organs are ejected: the AC, part of gonad tubules and the entire digestive system, except the cloaca [37], [38] (Fig. 1, 2). After evisceration, the holothurian retains the gonad, organs of the respiratory system (respiratory trees), the cloaca, and the intestinal mesentery, on which the digestive tube is located. The regeneration of all missing structures during summer months at a water temperature of 18–20 °C takes approximately 30 days [39], [31]. The regeneration process may be divided into 8 stages (Fig. 2). The restoration begins with the formation of a thrombus at the anterior end of the animal. During the first stage (one day after evisceration), the thrombus begins to be replaced by the extracellular matrix. Two to three days after evisceration (stage 2), a connective tissue thickening, the primordium of the AC, is formed at the anterior end of the holothurian. During the third stage (4–5 days after evisceration), a rod-like thickening, the anterior gut primordium, begins to grow from the AC backward along the torn edge of the mesentery. During the fourth stage (6–7 days after evisceration), this primordium elongates. At this time, the intestinal lining begins to form. It is formed at the expense of transdifferentiation of mesodermal cells [34]. Groups of mesodermal cells migrate from the mesenteric surface to the connective-tissue cord to form the luminal epithelium of the new intestine. The cell migration and, possibly, transdifferentiation are associated with the remodeling of the ECM of the gut primordium. During the fifth stage (8–10 days after evisceration), the posterior gut primordium becomes noticeable. It grows from the cloaca and extends along the mesenteric edge. During the sixth stage (12–14 days after evisceration), the main structures of the AC form, and the AC increases in size. The anterior gut primordium continues to grow backward along the mesenteric edge and reaches the middle of the body. During the seventh stage (16–18 days after evisceration), the anterior and posterior primordia unite to restore the entire gut, and during the eighth stage (25–30 days), the holothurians begin feeding.

In this paper we for the first time identified and partially characterized proteases in one member of the order Dendrochirotida. In example of *E. fraudatrix,* we described (for the first time in holothurians) the biochemical properties and substrate specificity of proteases expressed during regeneration. We propose that metalloproteinases play a key role in regeneration of the gut primordium in the holothurian *Eupentacta fraudatrix*.

## Materials and Methods

### Ethics Statement

No specific permits were required for the described field studies. Holothurians *Eupentacta fraudatrix* are unregulated widespread animals, which do not require specific protection. These animals were collected in the areas, which were not privately-owned or protected in any way. All procedures were conducted with a priority on animal welfare.

### Animals

The holothurian *Eupentacta fraudatrix* (Diakonov et Baranova) (Holothuroidea, Dendrochirotida) is widely distributed in coastal waters of the Sea of Japan in depths of 0.5 to 12 m. This is a small animal, whose maximum length reaches 9–10 cm. The study was conducted on sexually mature specimens collected in the Peter the Great Bay of the Sea of Japan. For experiments we chose animals that were about 5–7 cm long. Immediately after collection and during the experiments, the animals were kept in aerated sea water aquaria at 15–16 °C.

Evisceration was induced by injecting distilled water into the body cavity [37]. For biochemical analysis, holothurians were dissected at stages 1, 2, 4, and 6 of regeneration. The anterior primordium of the gut, together with a portion of the mesentery bearing the mesenteric hemal vessel, was cut out with scissors. The anterior region of the gut, together with a portion of the mesentery from uninjured animals, was used as a control. Ten to fifteen specimens were used at each stage and for the control.

In experiments studying the inhibition of metalloproteinases, we used 20 mM 1,10-phenanthroline in filtered sea water. A sample of dry 1,10-phenanthroline was first dissolved in 50 µl of ethanol and diluted in 5 ml of sea water. The resulting solution of 1,10-phenanthroline was injected into the body cavity at a dose of 0.05 ml/g of holothurian weight. In the control group, the animals were injected with the same volume of sea water containing 10 µl/ml of ethanol. To determine the stage of regeneration, animals from different days after evisceration were fixed with 4% formalin and dissected. Gut primordia were examined and photographed using a Leica EZ4D stereomicroscope.

For light microscopy, the animals were entirely fixed in Bouin's fixative [40] for 1–7 days at 4 °C, embedded in paraffin and cut into 6 µm slides. The sections were stained with hematoxylin and eosin afterstaining solution [40]. The resulting slides were examined and photographed using a Jenamed 2 (Carl Zeiss, Jena) light microscope equipped with a Nikon D1x digital camera.

### Materials

In this work, we used the protease inhibitors, 1,10-phenanthroline (P9375, Sigma) and phenylmethylsulfonyl fluoride (PMSF) (P7626, Sigma), and a cocktail of irreversible protease inhibitors containing AEBSF-HCl (an inhibitor of serine proteases), 4-amidinophenyl-methane sulfonyl-fluoride (APMSF-HCl) (an inhibitor of trypsin-like serine proteases), bestatin hydrochloride (an inhibitor of aminopeptidases), *trans*-epoxysuccinyl-L-leucylamido-(4-guanidino)butane (E-64) (an inhibitor of cysteine proteases).

The substrates used to reveal proteases on zymograms were gelatin from porcine skin (G8150, Sigma), collagen type I solution from rat tails (C3867, Sigma), and casein from bovine milk (C6554, Sigma). Other reagents for electrophoresis and zymography were obtained from Gerbu. Prestained molecular weight markers, SDS7B2, were obtained from Sigma. Solutions were prepared using ultrapure deionized Milli-Q water.

### Tissue Homogenates

Tissues were sampled from 10–15 animals and homogenized using an ultrasonic homogenizer 3 times for 5 s at 4 °C in 50 mM Tris-HCl buffer, pH 7.5, with 1% Triton X-100 or 1% SDS (laurylsulfate sodium salt), 1∶3 (w/v). In some experiments, protease inhibitors were added to the homogenization solution. The homogenate was incubated at 4 °C for 30 min and centrifuged for 10 min at 5000 rpm using a JA-20 rotor (Beckman). The supernatant (tissue extract) was collected and analyzed by zymography. To study the collagenase/gelatinase activity dynamics during gut regeneration, tissue samples were immediately frozen and stored at a temperature of -80 °C for no longer than 30 days.

### Zymographic Assay

Protease activity was assayed by direct zymography preceded by vertical electrophoresis (150 V, 2 h) of the tissue extract on 10% SDS-PAGE copolymerized with 0.1% gelatin, casein, or collagen I as the substrate. The samples were mixed with Laemmli buffer for electrophoresis (without β-mercaptoethanol and dithiothreitol (DTT)) to achieve an SDS concentration of 2.5%. After electrophoresis, the SDS was removed from the gels by several washes with 2.5% Triton X-100 in 50 mM Tris-HCl, pH 7.5. The zymograms were subsequently developed for 18 h at 30 °C in the same buffer with 5 mM CaCl_2_, 0,1 M NaCl and 0.02% NaN_3_.

It is known that echinoderm collagen denaturates at 25 °C–30 °C [41]. Thus, for determination of substrate specificity of proteases the electrophoresis and zymography were performed at 4 °C and 25 °C respectively.

In some experiments, the incubation buffer contained protease inhibitors (PMSF or 1,10-phenanthroline or cocktail of irreversible protease inhibitors). For gelatinolytic activity inhibition assays, the compounds were freshly solubilized in ethanol and diluted with Tris buffer used for developing the zymograms. For inhibition assays, the gel slab was then cut into slices corresponding to the lanes and put in different tanks containing the stated concentrations of inhibitors.

Gels were stained with 2.5% Coomassie Blue R-250 in 50% methanol and 10% acetic acid for 30 min and washed for 1–2 min in 30% methanol and 10% acetic acid. Unstained bands on the zymogram indicated the presence of proteases that degraded gelatin or collagen.

Densitometric analysis was performed using ImageQuant 5.2 and GrafPad Prism 3.0 computer programs after scanning the zymograms. Lytic activity was assessed by measuring the area of substrate hydrolysis in the lanes on an inverted image of the scanned gel. These densitometry values are here regarded as the levels of gelatinolytic activity. Activity was expressed either in arbitrary units (a.u., the integrated value of the intensity of all pixels in the spot minus the intensity of the background pixels) or as a percentage of the total protease activity of the entire sample in the lane measured on the given zymogram. Measurements of each sample were made three times in at least 3 different gels.

Prior to the experiments, we determined the linear portion of the relationship between the size and density of the inverted image of the lysis zone and the concentration of loaded protein. For quantitative electrophoresis and zymography, thirty micrograms of protein was loaded into the wells of the gels.

The concentration of protein in the homogenates was determined by the Bradford method. SDS was first added to samples containing Triton X-100 to a final concentration of 2% [42]. The protein concentration for each sample was calculated from a calibration graph. The calibration curve was plotted using serum albumin as a standard.

A mixture of prestained standard proteins with known molecular mass was used to determine the molecular mass of the proteases on the zymogram.

### Statistical Analysis

Results are presented as mean values and standard error (± SE). A comparative analysis of the obtained data was carried out using the Kruskal-Wallis ANOVA for unpaired statistics, with p≤0.05 as the threshold for statistical significance. In the analysis of the correlation of enzyme activity to substrate, the module of the correlation coefficient was considered high at a value greater than 0.75.

## Results

### Protease Extraction from Holothurian Tissues

**Figure 3:**
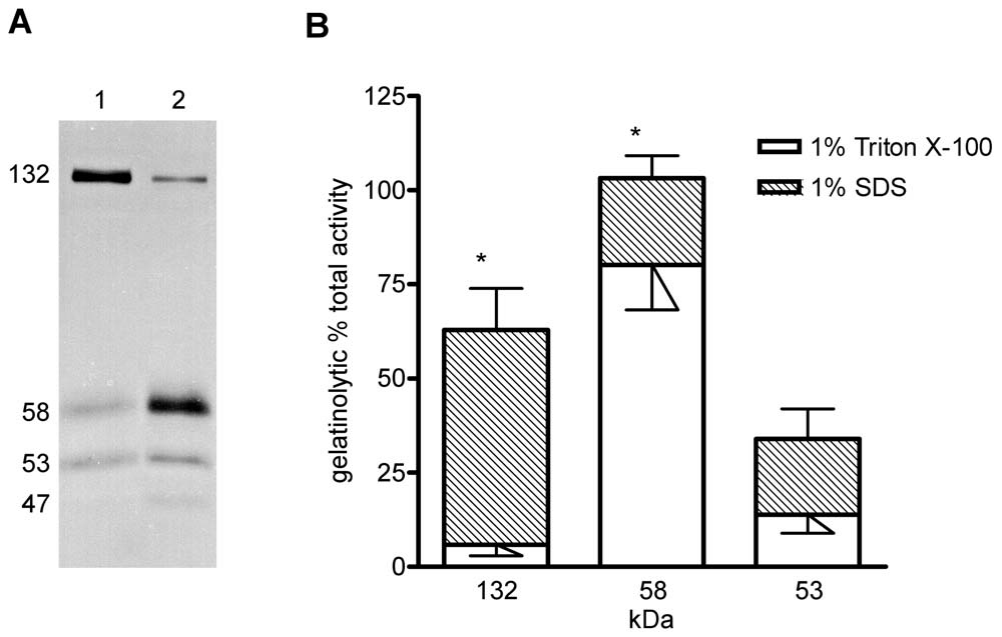
Gelatinolytic activity in the gut primordium. (A) Inverted image of zymography of the homogenates: lane 1, 1% Triton X-100 homogenate; lane 2, 1% Na-SDS homogenate. Thirty micrograms of the homogenate protein was resolved by 10% SDS-PAGE in the presence of 1 mg/ml gelatin. (B) Densitometric analysis of horizontally scanned lanes 1 and 2. Results represent the mean±SE of 9 different zymograms corresponding to 3 zymograms from each of the 3 different homogenates per experimental condition (*, high correlation score between gelatinase activity and used detergent, significant with p≤0.001).

Currently, there is no information on the properties of proteases from the holothurian *E. fraudatrix* in the literature; therefore, we preliminarily investigated the influence of different solubilizing agents on the effectiveness of enzyme extraction from tissue. For this purpose, gut primordia from the fourth stage of regeneration were used. At this stage, the MMP activity increases because the rudiments of the main organ systems are being formed and the remodeling of the extracellular matrix takes place [39], [38], [29], [35]. We used two homogenizing solutions, which contained detergents differing in their nature and strength of effect: a nonionic detergent, Triton X-100, and an ionic detergent, SDS. Irrespective of the extraction agent, gelatin zymography revealed four lytic zones, which corresponded to proteins showing proteolytic activity (Fig. 3A, Table 1). Their apparent molecular masses were 132, 58, 53 and 47 kDa. However, the gelatinolytic activity of the proteases with a molecular mass of 58 and 132 kDa depended on the detergent used (Fig. 3B). Thus, the 58 kDa protease activity was 3.5 times higher when extracted with Triton X-100, and the 132 kDa protease activity increased almost 10 times when SDS was used. The gelatinolytic activity of the 53 kDa protein was not dependent on the extraction detergent. We were unable to quantify the 47 kDa gelatinase activity by the methods used in this study because of the low lytic activity of this enzyme. Moreover, the lytic zones of all low molecular weight proteins were weakly expressed and had indistinct boundaries when using SDS for extraction. This made quantitative estimation of their lytic activity impossible. As a result, we extracted these proteases with Triton X-100 in subsequent experiments.

**Table 1:** Molecular masses of gelatin hydrolyzing proteins in the gut primordium of *Eupentacta fraudatrix.*

Molecular mass, kDa	SE	N
132	3	8
58	2	9
53	3	9
47	3	5

SE–standard error mean, N- number zymograms for analysis.

Because the experimental design included the collection and storage of tissue samples for 2–3 weeks, a number of experiments were conducted to determine the influence of sample storage conditions on the lytic activity and spectrum of the assayed proteases.

**Figure 4:**
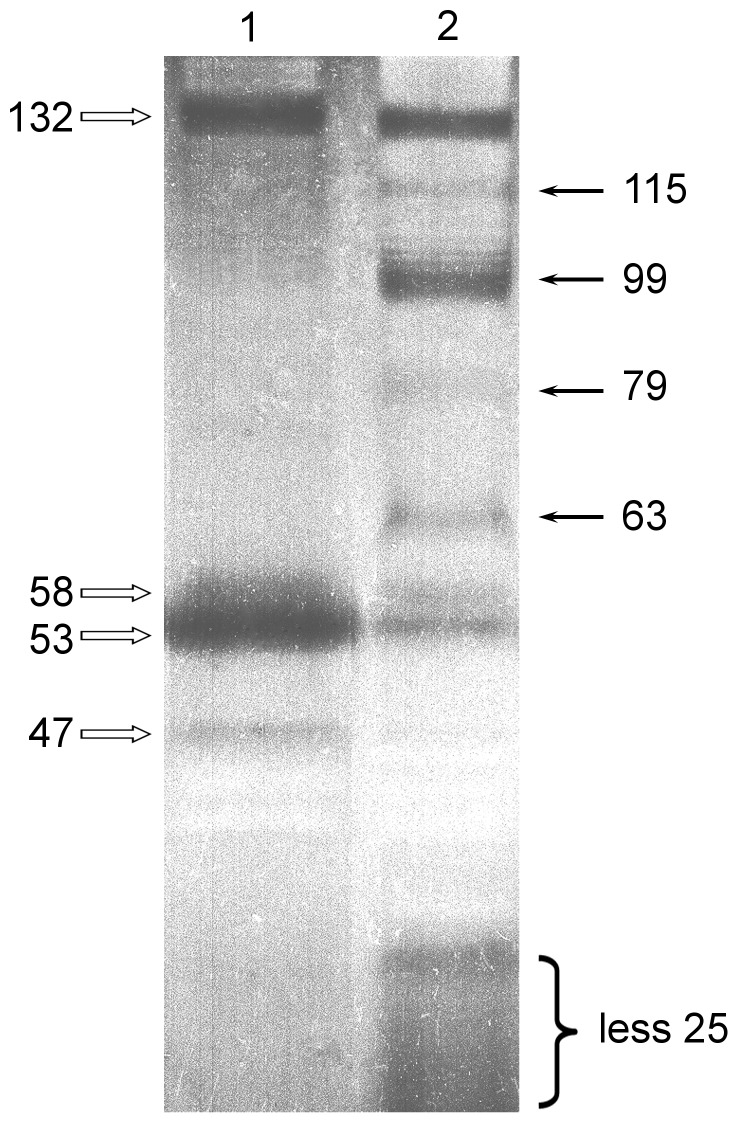
The effect of storage conditions on the composition of the gut primordium lytic bands. Inverted image of zymography of the homogenates: lane 1: homogenate isolated from a frozen tissue (sample 1); lane 2: frozen homogenate after 20 days of storage (sample 2). White arrows indicate lytic zones and molecular masses of the proteinases (kDa) from the freshly isolated homogenate of the gut primordium. Black arrows indicate new lytic zones and molecular masses of the proteinases (kDa) after thawing the homogenate. Images of gelatin zymograms (10% SDS-PAGE with 0.1% gelatin).

The gut primordia were cut out from twenty animals. Then, the material was divided into two portions. One portion was immediately frozen at −80 °C. Protein extraction from this portion was conducted several days later immediately prior to zymographic analysis (sample 1). The second portion of the material was used for the preparation of a Triton X-100 homogenate. A soluble protein fraction was extracted from this homogenate and frozen at −80 °C (sample 2). Gelatin gel zymography of both samples were made concurrently. The protease spectrum of sample 1 was identical to that of the freshly prepared extract (Fig. 4). In addition, on the zymogram of sample 2, there were several new lytic zones corresponding to proteins with molecular masses of 63, 79, 99, and 115 kDa (Fig. 4). The results indicate the possible dissociation of a high molecular weight protein during storage and defrosting of the Triton X-100 homogenate; therefore, in the subsequent experiments, tissue samples were frozen and stored at −80 °C and homogenized on the day that electrophoresis was conducted.

### Influence of Substrates on the Proteolytic Activity of the Proteases

**Figure 5:**
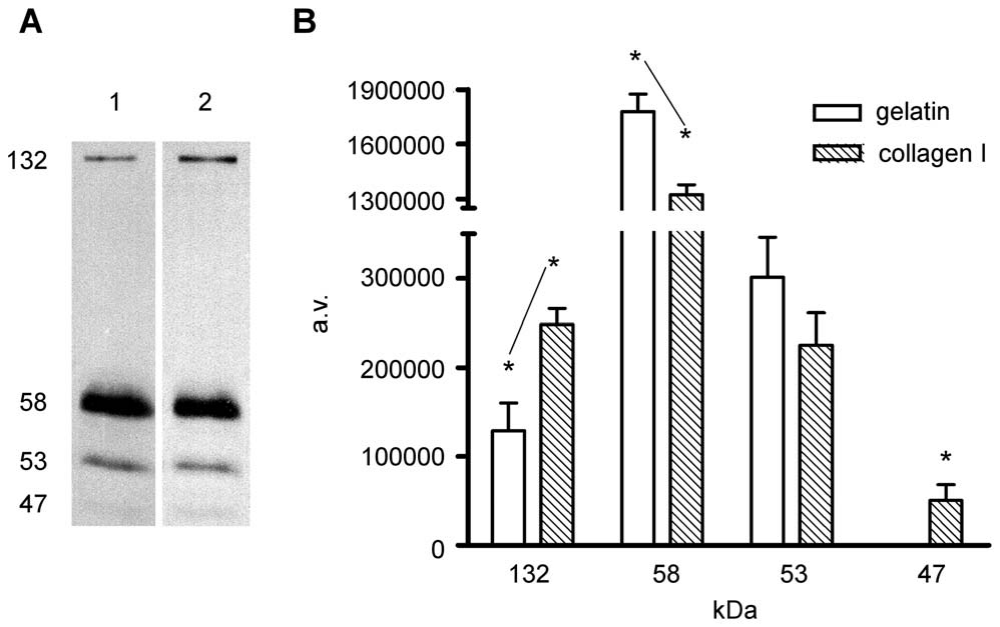
The effect of substrates on the protease activities of the gut primordium homogenate. (A) Inverted image of zymograms of the same homogenate with gelatin (1) and collagen I (2). Molecular masses (expressed in kDa) are indicated. (B) Densitometric analysis of the scanned bands. Adjusted volume (a.v.): proteinase activity represented the integrated intensity of all the pixels in the band, excluding the background. Results represent the mean±SE of 6 different zymograms corresponding to 3 zymograms from each of the 2 different homogenates per experimental condition (*–*, reliable differences between the compared values, p≤0.05).

We conducted a comparative analysis of the proteolytic activity of the same homogenate with respect to denatured collagen type A (gelatin), collagen type I, and casein on zymograms. Homogenates of gut primordia and the mesentery from the fourth stage of regeneration were used. None of the detected proteins showed proteolytic activity with respect to casein. On collagen type I zymograms, four lytic zones were revealed that corresponded to proteins with the same molecular masses as those observed on gelatin gels (Fig. 5). However, the 58 kDa protease activity was reliably low, compared to the gelatin zymogram, while the 132 kDa protease activity was reliably higher on the collagen type I zymogram. Moreover, the 47 kDa protease activity was consistently detected only on the collagen zymograms.

### Effect of Chelating and Thiol-Modifying Agents and Different Protease Inhibitors on Gelatinolytic Activity of Gut Primordium Proteases

Gut primordia and the mesentery from the fourth stage of regeneration were used in the experiments. All samples for electrophoresis were normalized for volume and protein content.

**Figure 6:**
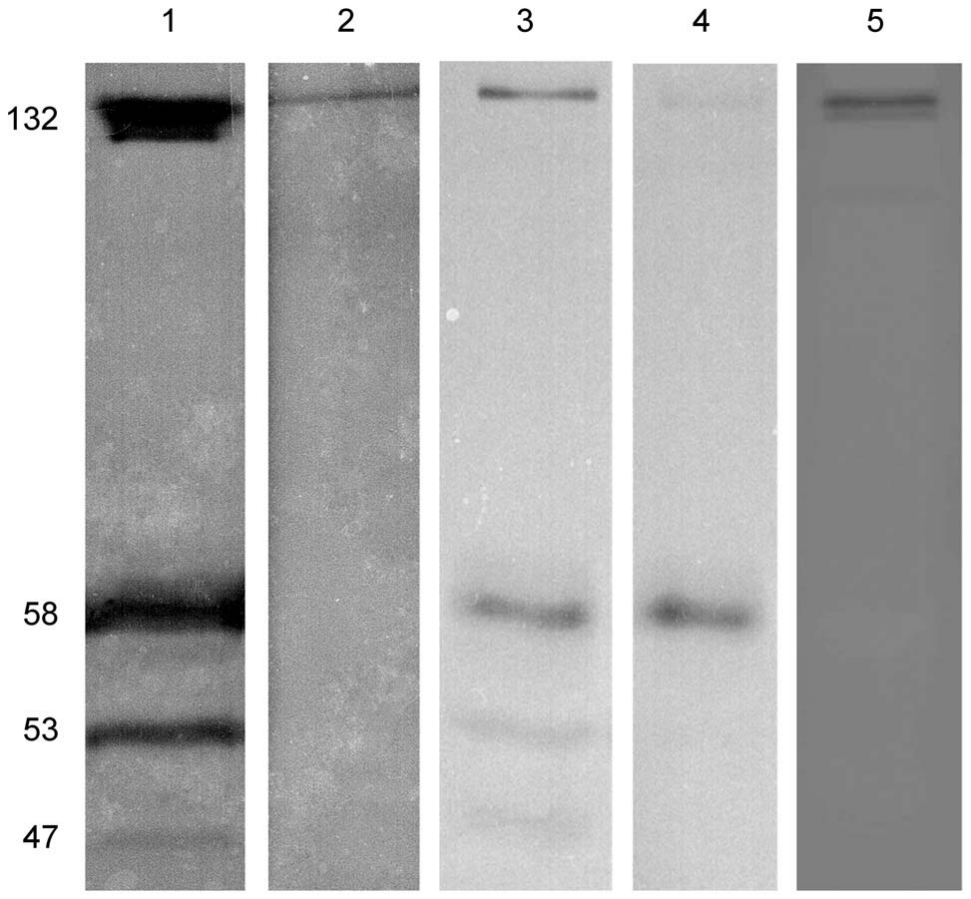
Inverted image of gelatin zymograms (10% SDS-PAGE) of the same homogenate from the gut primordium. (1) Gel incubated without agent; (2) with 2.5 mM of DTT; (3) with 1% Triton X-100 and 0.1 mM PMSF; (4) with 1% Triton X-100 and the protease inhibitor cocktail; (5) with 2 mM 1,10-phenanthroline. Thirty micrograms of total tissue protein was loaded in each lane. Molecular masses (expressed in kDa) are indicated.

The proteolytic activity of all enzymes was completely inhibited in the presence of 5 mM EDTA (Table 2). A similar effect was observed during the incubation of gels in a medium with a selective chelator of calcium ions, EGTA (5 mM). In this case, the addition of MgCl_2_ (55 mM) to the medium did not restore the enzyme activity. The thiol-modifying agent DTT (2.5 mM) completely inhibited the 47, 53 and 58 kDa protease activity and significantly reduced the lytic activity of the 132 kDa protein (Table 2, Fig. 6).

**Table 2:** Effect of low molecular inhibitors of enzymes on protease activity of gut primordium proteins of *Eupentacta fraudatrix.*

Inhibitor	Concentration of inhibitor	Inhibition, %			
		132 kDa	58 kDa	53 kDa	47 kDa
EDTA	5 mM	100	100	100	100
EGTA	5 mM	100	100	100	100
EGTA+MgCl_2_	5 mM+55 mM	100	100	100	100
DTT	2,5 mM	70	100	100	100

The effect of protease inhibitors on the activity of the proteolytic enzymes of *E. fraudatrix* was examined in two variants of an experiment. In one case, protease inhibitors were added to the homogenization buffer for protein extraction, and in the other case, protease inhibitors were added to the gel incubation medium for zymography. The irreversible serine protease inhibitor, PMSF, reduced the activity of all proteases in both variants of the experiment but did not completely inhibit it even at high (1 mM) concentrations (Fig. 6). An analogous scenario was observed with increasing the PMSF concentration in the gel incubation medium up to 10 mM (data are not shown). The cocktail of inhibitors completely inhibited the lytic activity of the 132, 53 and 47 kDa proteins in the gut primordium homogenate (Fig. 6), and only the 58 kDa protein was detectable on the gelatin zymogram from the two variants of the experiment. The specific inhibitor of zinc metalloproteinases, 1,10-phenanthroline, at a concentration of 2 mM significantly reduced the activity of the 132 kDa protein and completely inhibited the lytic activity of the 47, 53, and 58 kDa proteins (Fig. 6).

### Zymography for Gelatinolytic Activity Dynamics during Gut Regeneration

**Figure 7:**
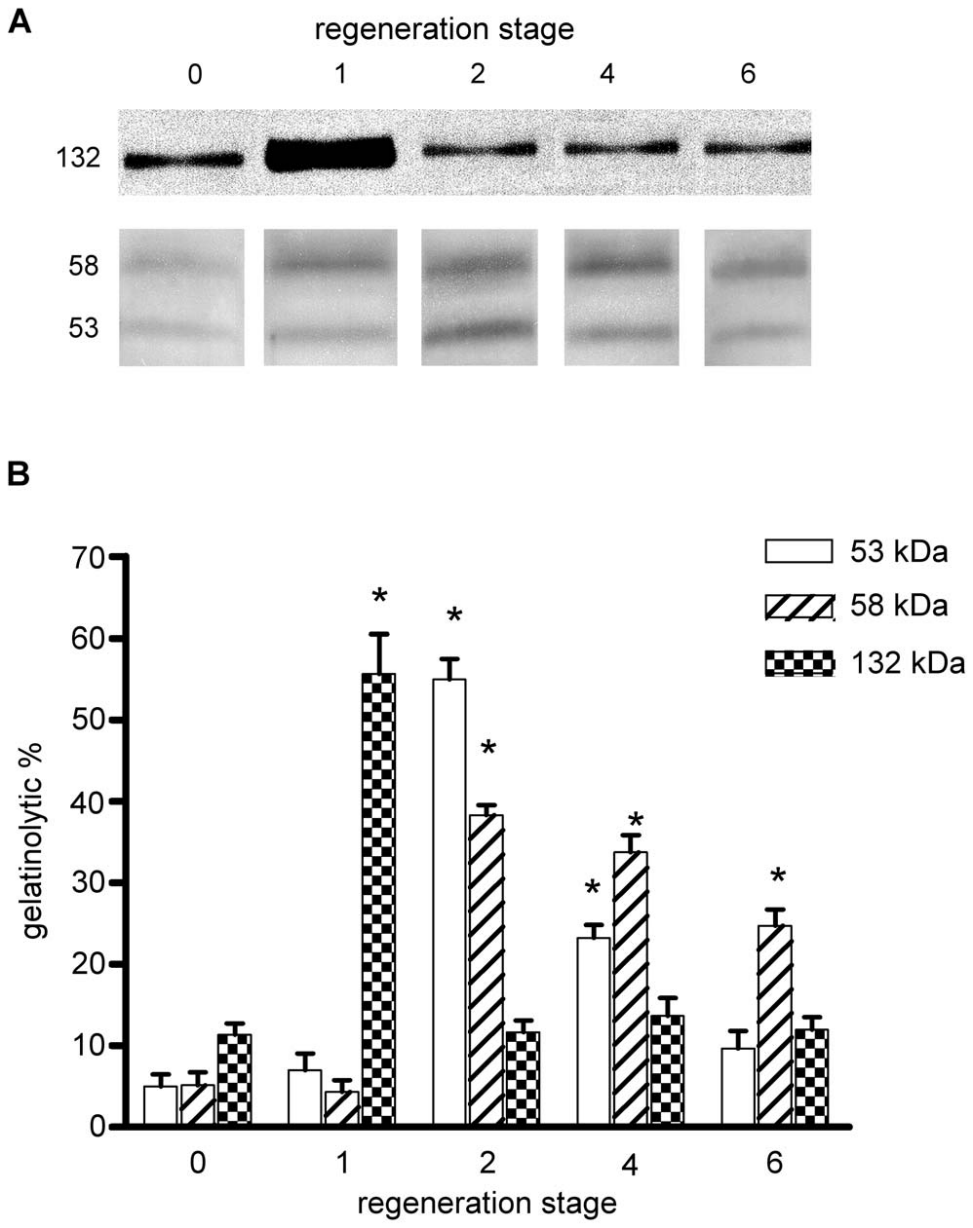
Gelatinolytic activity in the gut primordium during the process of regeneration. (A) Inverted image of zymograms of gut primordium homogenates from noneviscerated (day 0) and eviscerated (regeneration stages 1, 2, 4, and 6) animals. Molecular masses (expressed in kDa) are indicated. (B) Densitometric analysis of horizontally scanned bands. The percentage (%) represents the integrated intensity of all the pixels in the lytic band excluding the background as a percentage of the total value of horizontally scanned bands. Results represent the mean±SE of 6 different zymograms corresponding to 3 zymograms from each of the 2 different homogenates per experimental condition (*, p≤0.01 as compared with day 0, n = 6).

Samples of the intact gut (control) and gut primordia from stages 1, 2, 4, and 6 of regeneration were assayed. The composition of proteases was identical in the intact and regenerating gut and was not related to the regeneration stage. Densitometric analysis of the gelatin zymograms showed that lytic activity of proteases changed during the process of restoration (Fig. 7). Thus, in samples of gut primordium from the first stage of regeneration, the activity of the 132 kDa protein was statistically significantly increased, while the activity of the other proteins did not change substantially when compared to the control. During the second stage, the 132 kDa lytic activity decreased to the initial level and remained unchanged until the end of the experiment (sixth stage), while lytic activities of the 53 and 58 kDa proteases increased sharply. At the fourth stage, their activities remained high. Toward the end of the observation period (sixth stage), the 53 kDa gelatinase activity was decreased, while the 58 kDa protein activity, although decreased, was reliably higher than that of nonregenerating animals.

### Experimental Inhibition of Metalloproteinases with 1,10-Phenanthroline during Regeneration

**Figure 8:**
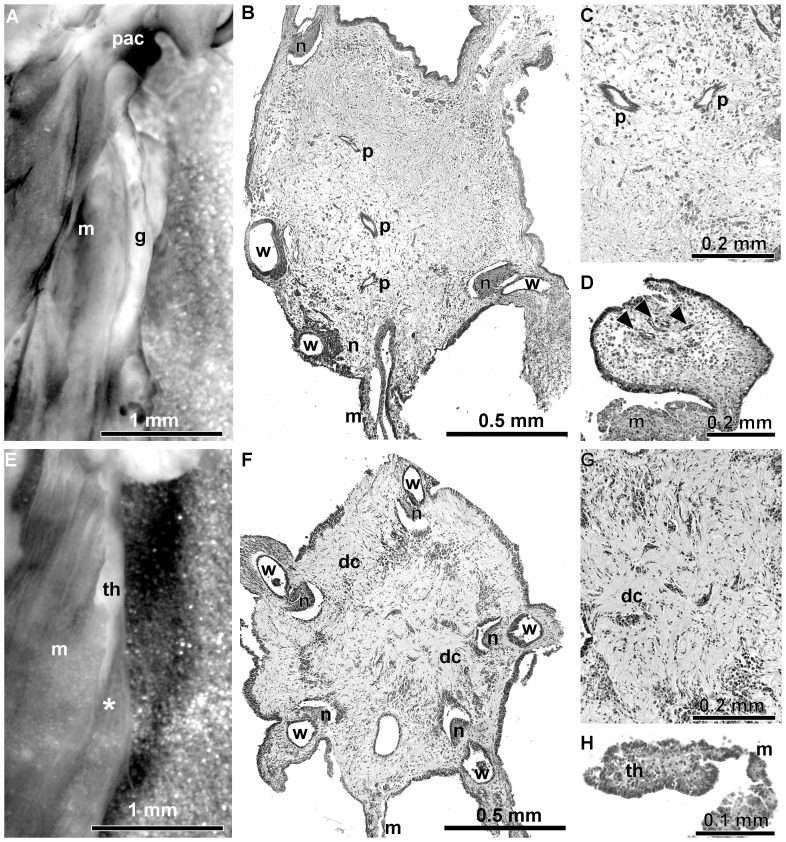
Morphology of internal organs in regenerating *Eupentacta fraudatrix* after inhibition of proteases with 1,10-phenanthroline for 14 days. (A) The general view of internal organs in a control animal. There are well developed anterior gut primordium and primordium of AC. (B) A paraffin section of AC primordium of a control animal. The primordium is composed of loose connective tissue containing numerous cells. There is primordium of pharynx comprising groups of cells with microcavities. (C) Part of AC primordium of a control animal at greater magnification. (D) A paraffin section of gut primordium of a control animal. The digestive epithelium already began developing (arrowheads). (E) The general view of internal organs in an experimental animal. There is only short thickening along the edge of mesentery. The asterisk indicates the end of the thickening. (F) A paraffin section of AC primordium of an experimental animal. The primordium is composed of dense connective tissue. The primordium of pharynx is absent yet. (G) Part of AC primordium of an experimental animal at greater magnification. (H) A paraffin section of gut primordium of an experimental animal. There is only small connective tissue thickening at the edge of mesentery. Abbreviations: dc, dense connective tissue; g, gut primordium; m, mesentery; n, radial nerve cord; p, primordium of the pharynx; pac, primordium of the AC; th, connective tissue thickening; w, radial water-vascular canal.

To study the role of proteases in gut regeneration, two experiments with 1,10-phenanthroline were conducted. In the first experiment, 20 animals were injected with 1,10-phenanthroline into the body cavity beginning at the second stage of regeneration when AC primordium began developing. The inhibitor was injected every day for 14 days. The control group of 20 holothurians was injected for the same period of time with sea water containing 10 µl/ml of ethanol. Afterward, 10 animals from each group were fixed and then dissected to study their regeneration stages. All control holothurians were found to be at the fourth stage of regeneration (Fig. 8A).

The sections through AC primordium showed that it mostly consisted of loose connective tissue with numerous cells located among the fibers of the latter (Fig. 8B, C). In the central part of the primordium there were lumens lined with cells; later on these cells would make up the lining of pharynx. Gut primordium represented a well-developed thickening along the edge of mesentery (Fig. 8D). Gut lining already began developing within the ECM of the thickening.

Phenanthroline-injected holothurians showed a delay in development, and all of them were found to be at stages 2 of regeneration (Fig. 8E). Sections through the AC primordium showed that it was mostly constructed of dense connective tissue (Fig. 8F, G). The latter was located on both the periphery of AC and in its central part. No gut primordium was present. There was only a small thickening at the edge of mesentery (Fig. 8H).

**Figure 9:**
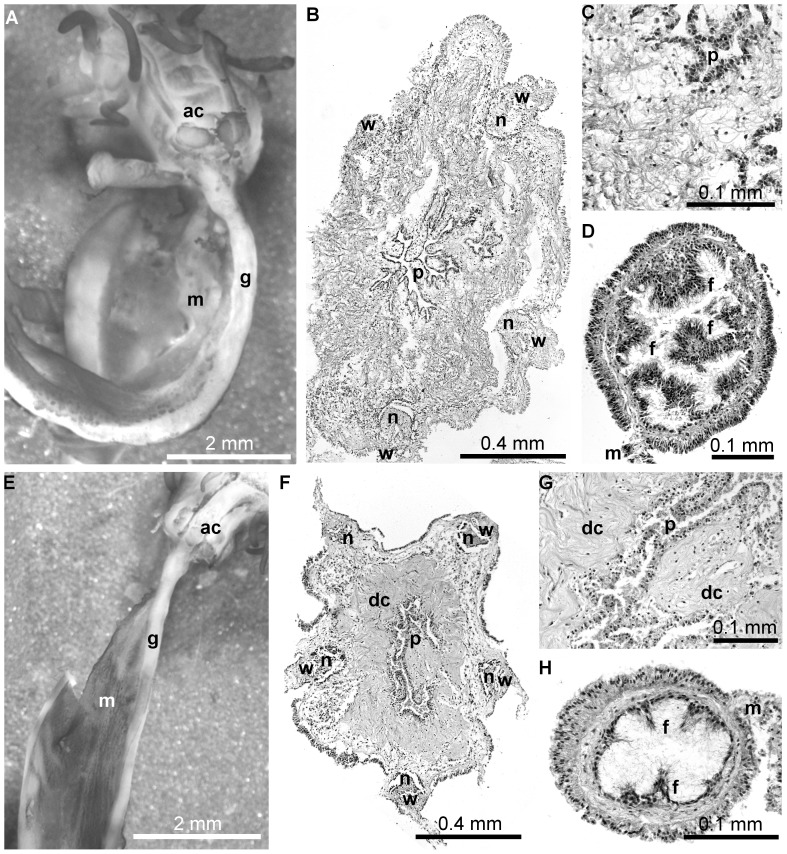
Morphology of internal organs in regenerating *Eupentacta fraudatrix* in 14 days after cancellation of 1,10-phenanthroline treatment. (A) The general view of internal organs in a control animal. There are well developed gut and AC. (B) A paraffin section of AC of a control animal. The AC is composed of loose connective tissue containing numerous cells. There is well-developed pharynx. (C) Part of AC of a control animal at greater magnification. (D) A paraffin section of gut of a control animal. The digestive epithelium develops high folds. (E) The general view of internal organs in an experimental animal. There are well developed gut and AC. (F) A paraffin section of AC of an experimental animal. The AC contains pharynx encircled by a layer of dense connective tissue. (G) Part of AC of an experimental animal at greater magnification. (H) A paraffin section of the gut of an experimental animal. The digestive epithelium forms small folds. Abbreviations: ac, aquapharyngeal complex; dc, dense connective tissue; f, fold of the digestive epithelium; g, gut; m, mesentery; n, radial nerve cord; p, pharynx; w, radial water-vascular canal.

The remaining animals from the control and experimental groups were maintained for another 14 days under normal conditions without an injection of 1,10-phenanthroline or water. At the end of this term (31 days after evisceration), these remaining animals were fixed and dissected to examine their stages of regeneration. Both the control and experimental animals developed a normal intestine. All control holothurians had good, developed internal organs corresponding to the eighth stage of regeneration (Fig. 9A).

Paraffin sections through AC of control animals showed well-developed pharynx epithelium (Fig. 9B, C). Pharynx walls were constructed of loose connective tissue infiltrated with numerous cells. The gut was well-developed (Fig. 9D). Intestinal epithelium made up numerous clearly pronounced folds. Apical parts of the cells contained secretory granules.

Phenanthroline-injected holothurians showed a delay in development, and all of them were found to be at the late seventh stage of regeneration (Fig. 9E). Paraffin sections through AC showed still retained areas of dense connective tissues (Fig. 9F, G). Despite this fact, pharyngeal epithelium was fairly well-developed. The structure of gut was similar to the normal one. Enterocytes contained numerous secretory granules. However, unlike the control animals, the folds of intestinal epithelium were worse developed (Fig. 9H).

In a second experiment, the test group (20 specimens) began to be injected with 1,10-phenanthroline at the first stage of regeneration. The inhibitor was injected every day for 11 days. The control group also consisted of 20 specimens, which were injected with sea water containing 10 µl/ml of ethanol in the body cavity on the same days. After 7 days of injection with 1,10-phenanthroline, the state of the test animals was already noticeably impaired. The body of these holothurians was inflated, ambulacral feet were retracted, and the animals were unattached to the wall of the aquarium. Ten experimental animals dissected the next day after the inhibitor injections ceased (12 days after evisceration) were found to be at the second stage of regeneration. These animals only had a small connective-tissue thickening at the anterior end and no gut primordium. Control animals dissected on the same day were found to be at the fifth stage of regeneration.

Four to five days after the inhibitor injections ceased, all animals of the test group died. The control holothurians developed normally and showed no deviation during regeneration.

## Discussion

In the present work, we detected proteins possessing collagenase/gelatinase activity in the digestive system tissues of the holothurian *E. fraudatrix* and investigated the expression of these proteins under normal conditions and during gut regeneration after evisceration. We detected 4 proteins with molecular masses of 132, 58, 53 and 47 kDa possessing lytic activity toward collagen type I and gelatin. None of the detected proteins hydrolyzed casein.

It is known that SDS destroys the fibrillar organization of collagen impregnated into the gel. Moreover, increasing temperature during electrophoresis or high (above 37 °C) temperature at zimographic assay also causes its denaturation. However, the method of collagen zymography is often used, in particular to reveal the activity of interstitial collagenase (MMP1) in mammals [43], [44]. It was shown that during zymography Triton X-100 replaces SDS and partially recovers the fibrillar organization of collagen making its available as a substrate for collagenase. Moreover collagen from sea urchins denaturates at 25 °C–30 °C [41]. In our experiments we tried to reduce the risk of collagen degradation and performed electrophoresis and zymography at 4° and 25 °C respectively. Thus, we believe the proteases of *E. fraudatrix* to have different substrate specificity. Under identical conditions, the 132 and 47 kDa proteins degraded collagen I more actively, while the 58 kDa protein was more active toward gelatin.

The ability of these proteases to degrade collagen and gelatin, in addition to a lack of catalytic activity with respect to casein, suggests that these proteins may function as extracellular proteases. The differences in proteolytic activity toward collagen and gelatin indicate that the proteins in these holothurians can have different substrate specificities and, possibly, different functions. Further studies using a range of different artificial substrates may assist in identifying these holothurian proteases.

Extracellular proteases with collagenase-gelatinase activity have been previously identified in other representatives of Echinodermata, such as sea urchins. Proteases were found in sea urchin embryos at different stages of development and in the peristomal connective tissue of adults [21], [45], [46], [47]. Some gelatinases detected in these animals were able to hydrolyze their own collagen and collagen type I from rat tails but were not active toward casein. One of the enzymes proved to be collagenase, whose main function is the reorganization of the embryo's hyaline layer [48]; the other two gelatinases (molecular masses of 57 and 50 kDa) are involved in the ECM remodeling during gastrulation [45]. In most cases, the molecular mass of gelatinases in sea urchins does not exceed 60 kDa. The exception is inducible gelatinases of the hyaline layer with a molecular mass from 90 to 117 kDa [49].

In the digestive tube tissue, we found a high molecular mass protease (132 kDa), which is generally not typical of extracellular proteases. The high molecular mass of this protease suggests that it may be a protein complex. Thus, the proenzyme of the gelatinase MMP-9, with a molecular mass varying from 91 to 96 kDa depending on the cell type, can form SDS-stable complexes with tissue inhibitors [50] or form tetramers and dimers with molecular masses of 225 and 130 kDa [51]. An extracellular gelatinase with a molecular mass of 41 kDa was found in the blastocoels of sea urchin embryos; however, in the native state, this protein forms a tetramer with a molecular mass of 160 kDa [52]. We found that the 132 kDa protein was resistant to SDS and showed the highest activity on the zymogram of the holothurian tissue homogenate solubilized in the presence of this detergent (Fig. 3). Furthermore, experiments with freeze-thawing of the gut rudiment homogenate detected the presence of proteins with molecular masses of 115, 79, 63 and less than 25 kDa that have collagenolytic activity, and these proteins may be the dissociation products of the 132 kDa protein.

The molecular mass of the other proteases in the gut of *E. fraudatrix* (58, 53 and 47 kDa) were comparable to those of intestinal gelatinases of the holothurian *H. glaberrima* [29]. The zymographic assay of this species detected 4 proteins with molecular masses of 59, 51, 45, and 43 kDa. On the basis of the inhibitor analysis, the authors considered these gelatinases to be MMPs.

Enzymes of the MMP family have several characteristic features: they hydrolyze one or several components of the extracellular matrix and basal laminae; contain zinc in the active center; belong to the calcium-dependent proteinases; are inhibited by chelators; and are secreted as proenzymes, which are activated by a number of proteinases and thiol-modifying agents [7]. We found that the proteases from *E. fraudatrix* hydrolyze different types of collagen and are calcium dependent because the activity of these enzymes was inhibited not only by EDTA but also by EGTA. Moreover, 1,10-phenanthroline completely inhibited the 58, 53 and 47 kDa gelatinase activity and significantly reduced the lytic activity of the 132 kDa protein. These findings suggest that these proteases have the biochemical properties of zinc metalloproteinases.

The partial inhibitory effect of the serine protease inhibitor PMSF on the proteases described in this work are most likely nonspecific. It is well known that PMSF is able to react with proteins and modify their biological functions; this observed effect does not involve the inhibition of the catalytic center of serine proteinases [53]. The incomplete inhibition of proteinase activity and the absence of a dose-dependent effect at different concentrations of PMSF (0.1 mM and 1 mM) confirm that the effect of this inhibitor on the holothurian *E. fraudatrix* proteases is nonspecific.

It is well known that 1,10-phenanthroline can inhibit not only MMPs but also aminopeptidases [54]. In our experiments, the cocktail consisting of irreversible inhibitors of serine-, cysteine-, and aminopeptidases completely inhibited the 132, 53, and 47 kDa protein activity but was not effective against the 58 kDa protease. It is unlikely that the 132, 53 and 47 kDa proteins are cysteine proteinases; otherwise, DTT would have had a stimulatory effect on the lytic activity of these proteins. It is possible that these proteins have aminopeptidase activity. Thus, with regard to the sensitivity to the cocktail of inhibitors and 1,10-phenanthroline, only the 58 kDa metalloproteinase is similar to MMPs of vertebrates. Based on molecular mass, this protein likely corresponds to the MMP from the regenerating gut of the holothurian *H. glaberrima* with a molecular mass of 59 kDa.

A characteristic feature of typical MMPs is the activation of the proenzyme by thiol-modifying agents, which destabilizes the bond between the zinc ion of the active site and the cysteine residue, thus leading to the chemical activation of the proenzyme [12], [55]. In our experiments, DTT completely inhibited the lytic activity of all low molecular weight proteases. This means that S–S bonds obviously play certain role in maintaining the lytic activity of proteases in *E. fraudatrix*, which is also characteristic of enzymes of MMP family.

In holothurians, the basis for gut regeneration after evisceration is provided by the restructuring of the extracellular matrix of the intestinal mesentery [29], [30], [31], [34], [56], [57], [58]. The primordium of the new intestine is formed as a connective-tissue cord along the gut mesentery edge. Subsequently, cells migrate inside the cord to form the intestinal lining. Cell migration and the formation of the basal laminae involve the participation of enzymes that restructure the ECM. The activity of proteases and MMPs in holothurians changes during the process of regeneration [29], [35], present work. The highest activity was observed during the formation of the connective-tissue primordium and intestinal lining. During this period, the amount of fibrous collagen and laminin are decreased [29]. Inhibition of protease activity exerts a marked effect on regeneration.

During the studies of MMPs functions different substances are often used that inhibit the activity of these enzymes. One of the most commonly used agents is 1,10-phenanthroline [59], [60], [29], [61], [62], [63]. The inhibiting effect of 1,10-phenanthroline is due to its capability to bind zinc ions of the active center of MMP. A drawback of this agent is its capability to bind also other bivalent ions, in particular calcium and magnesium. In this connection, the observers should naturally take certain care during interpretation of the results of experiments with 1,10-phenanthroline *in vivo*. A specific feature of holothurians in particular and all echinoderms in general is that their coelomic fluid is similar in composition to seawater [64]. The seawater contains calcium and magnesium ions in high concentrations (approximately 10 and 55 mM respectively), thus we believe that the effects of 1,10-phenanthroline on regeneration in *E. fraudatrix* indeed are due to binding of zinc ions and inactivation of proteases.

In the holothurian *E. fraudatrix*, this effect was dependent on the time when 1,10-phenanthroline injections commenced. When metalloproteinases were inhibited at the second stage of regeneration, the restoration rates were decreased. However, such an effect proved to be reversible, and when inhibition ceased, the regeneration was recovered. These results are consistent with the previously reported data on the holothurian *H. glaberrima* [29]. In this species 1,10-phenanthroline causes retardation of gut development. The ECM remodeling is obviously inhibited, as much collagen is retained in mesentery and the primordium of digestive tube. In *E. fraudatrix* after treatment with 1,10-phenanthroline the primordium of AC and pharynx also consist of dense connective tissue that obviously contains greater amount of collagen. The dense connective tissue is retained in the organs even in 14 days after the cancellation of the blocker agent. The data on both holothurian species show that proteases, which were present in the mesentery and gut primordium, were likely involved in collagen degradation. Reduction in fibrous collagen allows the cells to migrate within the extracellular matrix; therefore, the inhibition of the proteases retards the regeneration process in holothurians.

In *E. fraudatrix* when protease activity is inhibited at the first stage, regeneration is completely abolished, and the animals die, suggesting that early activation of the proteases is crucial for triggering the regenerative process in holothurians. The main events in this period are the removal of degraded and injured tissue and the cleaning of the wound [38]. The inhibition of the activity of proteases could probably reduce the rate of migration of immune cells (coelomocytes) toward the wounded area. In echinoderms the great role in thrombus formation and immune response is played by spherulocytes [38], [65], [66], [67]. Their cytoplasm comprise numerous granules that can contain different biologically active substances, in particular lectins [66], [68]. Decreased activity of proteases reduces both the infiltration of wounded area with coelomocytes and immune response, which could provoke the activation of pathogenic microorganisms and the death of the animals.

The other reason of death of the holothurians after 1,10-phenanthroline injection at the first stage could be inhibition of regeneration process. It is known that early expression of MMP is required for normal wound repair [3], [69], [70], [71], [72]. In particular, different types of MMP take part in remodeling of basal lamina and initiation of cell migration and proliferation [73], [72]. During the regeneration of limbs in amphibians and insects, wound epithelization and innervation provide a specific environment, that induces the formation of blastema [3], [69], [74], [71]. FGF-signaling and MMP activities are supposed to play significant roles in the formation of appropriate environment and activation of regeneration. In the newt *Notophthalmus viridescens* the inhibition of MMP in some cases results in development of a defective limb; in other cases no regeneration happens at all and just a scar is formed in the wounded area [70]. Obviously, in *E. fraudatrix* the inhibition of proteases at the first stage suppresses the development of appropriate environment and activation of particular mechanisms that initiate the process of regeneration. In this case there is no regeneration of the AC and the entire anterior end of the animal (which is actually the “head region”), thus the holothurian would necessarily die.

Thus, four proteases identified in the gut tissue of the holothurian *E. fraudatrix* were determined to be zinc metalloproteinases. These proteases differ in molecular mass, sensitivity to inhibitors, and activity during gut regeneration. The metalloproteinases share the ability to cleave the constituent proteins of the extracellular matrix, which determines their functions in the organism. These metalloproteinases likely play a crucial role in holothurian regeneration, and their inhibition retards or abolishes the restoration process. Among these metalloproteinases, only the 58 kDa metalloproteinase is similar to MMPs of vertebrates. The 132, 53 and 47 kDa proteins may have aminopeptidase activity; however, further study is needed to elucidate the exact nature of these enzymes.

The holothurian *E. fraudatrix,* is, in our opinion, a convenient model object to study ECM remodeling during regeneration and the role of metalloproteinases in this process. Unlike holothurians of the order Aspidochirotida, in *E. fraudatrix* we revealed the natural process of transdifferentiation, when mesodermal cells (peritoneocytes and myoepithelial cells) give rise to endodermal gut epithelium [34]. The new gut lining is formed at the expense of migration of cells from the surface of primordium into its inner part. Metalloproteinases obviously play important role in the migration of mesodermal cells and their transdifferentiation. ECM and different MMP are known to play significant role in cell transformation of vertebrates [75], [76], [77], [78]. The studies of this process in *E. fraudatrix* could clarify the natural mechanisms of transdifferentiation and the roles of ECM remodeling and proteases in this process.
